# Autofluorescence bronchoscopy findings of pseudoprogression after neoadjuvant chemo-immunotherapy in squamous cell lung cancer: A case report

**DOI:** 10.1016/j.rmcr.2026.102388

**Published:** 2026-02-20

**Authors:** Himeko Mori, Yasuharu Sekine, Keisuke Kuroda, Sho Ueda, Yusuke Saeki, Naohiro Kobayashi, Hideo Ichimura, Noriko Takemura-Kobayashi, Daisuke Matsubara, Yukio Sato

**Affiliations:** a Department of Thoracic Surgery, University of Tsukuba, Tsukuba, Japan; b Department of Diagnostic Pathology, Institute of Medicine, University of Tsukuba, Ibaraki, Japan

**Keywords:** Autofluorescence bronchoscopy, Pseudoprogression, Neoadjuvant chemo-immunotherapy, Non-small cell lung cancer, Squamous cell carcinoma

## Abstract

In recent years, multiple clinical trials have demonstrated the efficacy of neoadjuvant chemotherapy (NAC) combined with immune checkpoint inhibitors (ICI) for stage II–III non-small cell lung cancer (NSCLC). Pseudoprogression, a phenomenon in which activated lymphocytes infiltrate tumors and cause transient enlargement, can complicate post-treatment assessment. We report a case of stage III squamous cell carcinoma in the right lower lobe with endobronchial spread treated with NAC plus ICI. Autofluorescence imaging (AFI) bronchoscopy after treatment showed further autofluorescence attenuation despite no residual malignancy, consistent with pseudoprogression. Histopathology revealed infiltration of CD4^+^, CD8^+^ lymphocytes, and CD68^+^ macrophages without cancer cells. This case highlights that autofluorescence attenuation after immunotherapy may reflect pseudoprogression rather than residual tumor, emphasizing the importance of re-biopsy before surgical decision-making.

## 1 Introduction

In recent years, multiple clinical trials have shown the efficacy of neoadjuvant chemotherapy (NAC) combined with immune checkpoint inhibitors (ICI) for stage II-III non-small cell lung cancer (NSCLC) [1,2], which has drastically changed the strategy for the treatment of locally advanced lung cancer. Pseudoprogression is a phenomenon in which activated lymphocytes infiltrate the tumor and cause an inflammatory response, resulting in tumor enlargement during immunotherapy, despite the absence of true cancer progression [3,4]. However, attention must be paid to the possibility of pseudoprogression, which can occur in 3.4% to 6.7% of NSCLC cases following immunotherapy and may compromise evaluation of treatment effects after NAC [5]. If pseudoprogression is misinterpreted as true tumor progression, it could lead to changes in treatment strategy, including the abandonment of surgery.

NAC combined with ICI is indicated not only for cases with lymph node or chest wall involvement but also for those with endobronchial spread of carcinoma. Autofluorescence imaging (AFI) with bronchoscopy is effective for evaluating the endobronchial spread of NSCLC. We present a case of stage III lung squamous cell carcinoma originating in the right lower lobe with endobronchial spread to the intermediate bronchus up to the second carina, treated with NAC combined with ICI. Bronchoscopic observation with AFI after NAC combined with ICI revealed pseudoprogression, with further attenuation of autofluorescence at the site of endobronchial spread, despite biopsy confirming no malignancy. The patient subsequently underwent successful right lower lobectomy.

## 2 Case presentation

**Fig. 1:**
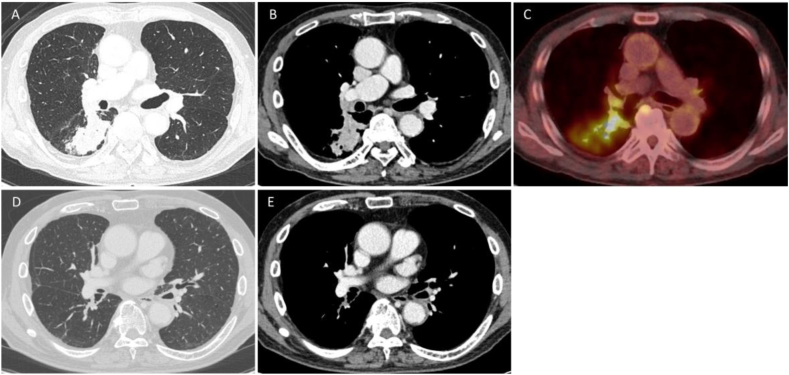
A and B: Chest computed tomography (CT) before neoadjuvant chemo-immunotherapy showing a 50-mm solid tumor in the right S6, a swollen #11s lymph node, and obstructive pneumonia peripheral to the tumor. C: Positron emission tomography (PET) before neoadjuvant chemo-immunotherapy. D and E: Chest CT after neoadjuvant chemo-immunotherapy showing near-complete disappearance of the tumor.

**Fig. 2:**
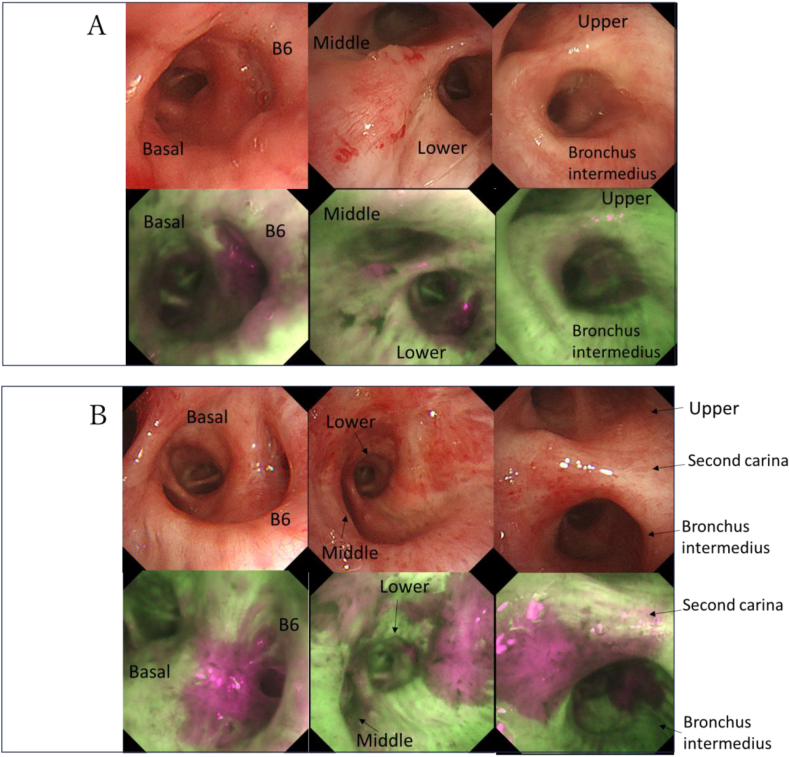
A: Bronchoscopy before chemo-immunotherapy. From left to right: bifurcation of B6 and the basal segment, bifurcation of the middle and lower lobes, and bifurcation of the upper lobe and bronchus intermedius. Autofluorescence imaging (AFI) shows discontinuous autofluorescence attenuation from B6 to the basal branch, the middle branch at the middle-lower lobe bifurcation, and the proximal bronchus intermedius. B: Bronchoscopy after chemo-immunotherapy, showing the same locations as Fig. 2A. AFI demonstrates more prominent autofluorescence attenuation at the second carina, proximal bronchus intermedius, and middle-lower lobe bifurcation compared with baseline.

A 78-year-old man with chronic obstructive pulmonary disease, hypertension, diabetes, and a heavy smoking history presented with hemoptysis and cough. Chest computed tomography (CT) revealed a 50-mm solid mass in the right S6 segment, accompanied by obstructive pneumonia and an enlarged interlobar lymph node (Fig. 1A and B). Positron emission tomography (PET) showed high fluorodeoxyglucose (FDG) uptake in the S6 tumor (SUVmax 14.65) and moderate FDG uptake in the interlobar lymph node (Fig. 1C). Fig. 2A shows the bronchoscopy findings before neoadjuvant chemo-immunotherapy. AFI showed continuous autofluorescence attenuation from B6 to the basal branch, the middle branch at the middle-lower lobe bifurcation, and the proximal bronchus intermedius. Bronchoscopic biopsy revealed tumor occluding B6, identified as keratinizing squamous cell carcinoma. Mapping biopsy confirmed squamous cell carcinoma invasion in all areas of autofluorescence attenuation. Therefore, the disease was staged as cT3N1M0, cStage IIIA (TNM 8th edition), for which right sleeve middle and lower lobectomy was indicated. Comprehensive genomic profiling and programmed death-ligand 1 (PD-L1) assays showed no driver mutations and no PD-L1 expression.

**Fig. 3:**
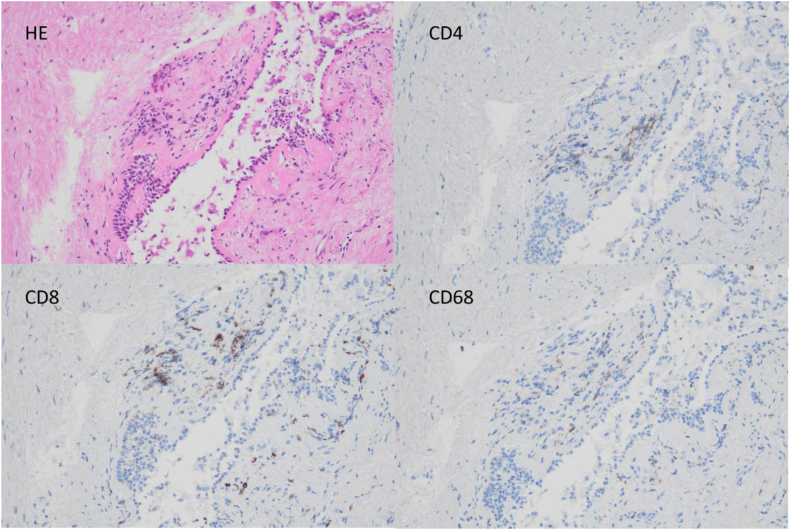
Histology of bronchoscopic biopsy specimen from the tracheal bifurcation. The bronchial epithelium is preserved, with inflammatory cell infiltration in the subepithelial interstitium. No loss or replacement of the bronchiolar epithelium by inflammatory cells is observed. The CD4/CD8 ratio is 0.89.

**Fig. 4:**
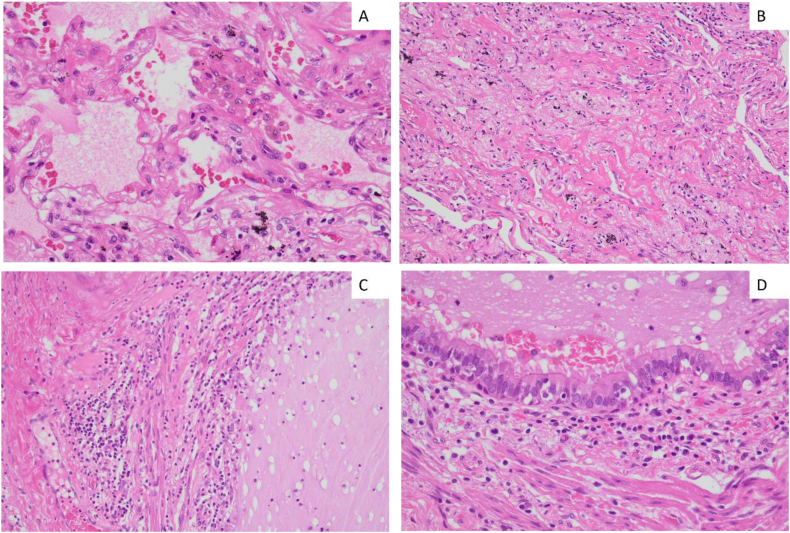
Histology of the surgical specimen. (A) Exudate accumulation in alveoli with red blood cell leakage and hemosiderin-laden macrophages. (B) Localized small-lymphocyte infiltration and alveolar collapse without prominent exudate; fibroblast formation is also observed. (C) Loss of bronchial epithelium with exposed subepithelial stroma, accompanied by inflammatory cell infiltration. (D) Focal inflammatory cell infiltration in the subepithelial interstitium without bronchiolar epithelial involvement. CD4^+^, CD8^+^, and CD68^+^ cells are mixed around the bronchi, with the number of CD68^+^ cells slightly less than or equal to that of CD4^+^ and CD8^+^ T lymphocytes. The CD4/CD8 ratio is 1.03.

Considering the predicted postoperative pulmonary function (vital capacity: 1616 mL [55.6%], forced expiratory volume in 1 second: 1136 mL [51.2%]), we planned to introduce NAC combined with ICI to reduce the extent of resection. The patient received three cycles of neoadjuvant chemo-immunotherapy consisting of carboplatin (AUC 5), paclitaxel (200 mg/m^2^), and nivolumab (360 mg), administered every three weeks. Post-neoadjuvant CT showed marked tumor shrinkage with resolution of obstructive pneumonia, although bronchial wall thickening around the B6 bronchus persisted. These findings were consistent with a partial response (PR) (Fig. 1D and E). Bronchoscopy after NAC combined with ICI showed further autofluorescence attenuation at the second carina, extending through the bronchus intermedius to the lower lobe bronchus, compared with pretreatment findings (Fig. 2B). All biopsy specimens from autofluorescence-attenuated areas were negative for malignancy and showed clusters of small lymphocytes (CD4^+^, CD8^+^) and CD68^+^ macrophages in the subepithelial layer (Fig. 3). Based on these pathological findings, the lesions spreading to the second carina were considered to have regressed. The patient therefore underwent right lower lobectomy with lymph node dissection. Intraoperative frozen-section analysis of the bronchial stump revealed no residual cancer cells. The final pathological diagnosis was ypT0N0. Notably, bronchial stromal areas exhibited focal lymphocytic clusters (CD4^+^, CD8^+^) and macrophages (CD68^+^), resembling the pathological features observed in bronchoscopic biopsies from areas of autofluorescence attenuation after NAC combined with ICI (Fig. 4).

## 3 Discussion

We reported a case showing pseudoprogression on bronchoscopy with AFI, despite disappearance of cancer cells following NAC combined with ICI. This case is notable because further autofluorescence attenuation on AFI was observed in areas where the tumor had pathologically regressed. AFI is based on the principle that normal bronchial mucosa emits strong green autofluorescence when excited by blue-violet light (420-460 nm), primarily due to collagen and elastin. In contrast, dysplastic or cancerous tissue shows attenuated fluorescence from epithelial thickening and increased blood content, appearing as magenta areas [6,7]. Therefore, non-malignant changes such as epithelial thickening, stromal inflammation, or vascular proliferation may also appear magenta [6]. Autofluorescence attenuation has also been reported at non-malignant sites, including airway complications after lung transplantation, tuberculosis-associated bronchial ulcers, and tracheobronchial amyloidosis [[8], [9], [10]].

ICI can occasionally induce pseudoprogression, typically characterized by transient tumor enlargement on X-ray or CT imaging. Although the mechanism is not fully understood, it is thought that inflammation from activated CD4^+^ and CD8^+^ T cells, macrophages, and other immune cells causes edema and bleeding within the tumor, leading to apparent tumor enlargement [11]. Although histopathological features of pseudoprogression are rarely reported, infiltration of activated lymphocytes (CD4^+^, CD8^+^) and macrophages (CD68^+^) is characteristic [[12], [13], [14]]. In our case, infiltration of activated lymphocytes and macrophages in the bronchial epithelium was considered to attenuate autofluorescence in the area previously invaded by the tumor.

An important question raised by this case is why pseudoprogression occurred exclusively in the endobronchial lesion despite marked shrinkage of the primary parenchymal tumor on CT. One possible explanation is that the bronchial mucosa and subepithelial layer contain a rich lymphatic and vascular network, which may facilitate localized immune cell infiltration following ICI. Another explanation is that conventional pseudoprogression detected by CT is typically a volume-based phenomenon, while AFI may be able to detect subtle immunotherapy-induced inflammatory changes at the tissue or mucosal level that are insufficient to produce detectable enlargement on CT. Such changes in the endobronchial mucosa may have been insufficient to produce detectable enlargement on CT, resulting in pseudoprogression limited to the endobronchial lesion.

To the best of our knowledge, this is the first report to demonstrate pseudoprogression on AFI bronchoscopy in squamous cell carcinoma treated with NAC combined with ICI. Importantly, recognition of endobronchial-only pseudoprogression has clinical implications even in institutions without autofluorescence imaging. In patients receiving neoadjuvant immunotherapy, apparent progression or persistence of endobronchial lesions on conventional bronchoscopy may not necessarily indicate residual malignancy. Awareness of this phenomenon may help prevent premature abandonment of surgical treatment or unnecessary extension of resection, underscoring the importance of repeat biopsy and careful pathological confirmation.

## 4 Conclusion

We report a case of pseudoprogression detected by autofluorescence imaging after neoadjuvant chemo-immunotherapy for squamous cell lung carcinoma. This case highlights that autofluorescence attenuation after immunotherapy may reflect pseudoprogression and suggests that pseudoprogression can occur exclusively within endobronchial lesions, even when the primary tumor shows an apparent radiological response.

## CRediT authorship contribution statement

**Himeko Mori:** Writing – original draft, Methodology, Data curation, Conceptualization. **Yasuharu Sekine:** Writing – review & editing, Data curation, Conceptualization. **Keisuke Kuroda:** Writing – review & editing. **Sho Ueda:** Writing – review & editing. **Yusuke Saeki:** Writing – review & editing. **Naohiro Kobayashi:** Writing – review & editing. **Hideo Ichimura:** Writing – review & editing. **Noriko Takemura-Kobayashi:** Writing – review & editing, Data curation. **Daisuke Matsubara:** Writing – review & editing. **Yukio Sato:** Writing – review & editing, Supervision.

## Ethical approval

Ethical approval was waived because this study reports a single case with informed consent.

## Consent

Written informed consent was obtained from the patient for publication of this case report and accompanying images.

## Funding

This research did not receive any specific grant from funding agencies in the public, commercial, or not-for-profit sectors.

## Declaration of competing interest

The authors declare no conflicts of interest.

## References

[1] Spicer J.D., Garassino M.C., Wakelee H. (2024). Neoadjuvant pembrolizumab plus chemotherapy followed by adjuvant pembrolizumab compared with neoadjuvant chemotherapy alone in patients with early-stage non-small-cell lung cancer (KEYNOTE-671): a randomised, double-blind, placebo-controlled, phase 3 trial. Lancet.

[2] Forde P.M., Spicer J., Lu S. (2022). Neoadjuvant nivolumab plus chemotherapy in resectable lung cancer. N. Engl. J. Med..

[3] Ferrara R., Caramella C., Besse B., Champiat S. (2019). Pseudoprogression in non-small cell lung cancer upon immunotherapy: few drops in the ocean?. J. Thorac. Oncol..

[4] Fujimoto D., Yoshioka H., Kataoka Y. (2019). Pseudoprogression in previously treated patients with non-small cell lung cancer who received nivolumab monotherapy. J. Thorac. Oncol..

[5] Park H.J., Kim K.W., Pyo J. (2020). Incidence of pseudoprogression during immune checkpoint inhibitor therapy for solid tumors: a systematic review and meta-analysis. Radiology.

[6] He Q., Wang Q., Wu Q., Feng J., Cao J., Chen B.Y. (2013). Value of autofluorescence imaging videobronchoscopy in detecting lung cancers and precancerous lesions: a review. Respir. Care.

[7] Sutedja T.G., Codrington H., Risse E.K. (2001). Autofluorescence bronchoscopy improves staging of radiographically occult lung cancer and has an impact on therapeutic strategy. Chest.

[8] Tezuka T., Inayama M., Suzue R., Miyamoto K., Haku T. (2020). A tuberculous bronchial artery aneurysm with abnormal findings on autofluorescence imaging bronchoscopy. Intern. Med..

[9] Mendogni P., Carrinola R., Gherzi L. (2020). Usefulness of autofluorescence bronchoscopy in early diagnosis of airway complications after lung transplantation. Sci. Rep..

[10] Uchimura K., Nemoto K., Manabe T., Yatera K. (2021). Emerald sign for diagnosing tracheobronchial amyloidosis. Intern. Med..

[11] Zhou L., Zhang M., Li R., Xue J., Lu Y. (2020). Pseudoprogression and hyperprogression in lung cancer: a comprehensive review of literature. J. Cancer Res. Clin. Oncol..

[12] Tanizaki J., Hayashi H., Kimura M. (2016). Report of two cases of pseudoprogression in patients with non-small cell lung cancer treated with nivolumab-including histological analysis of one case after tumor regression. Lung Cancer.

[13] Masuhiro K., Shiroyama T., Nagatomo I., Kumanogoh A. (2019). Unique case of pseudoprogression manifesting as lung cavitation after pembrolizumab treatment. J. Thorac. Oncol..

[14] Meoni G., Decarli N.L., Benucci M., Raspanti C., Ribecco A.S. (2020). Pseudoprogression in lung cancer: a case report. Explor Target Antitumor Ther..

